# Towards fair health policies for migrants and ethnic minorities: the case-study of ETHEALTH in Belgium

**DOI:** 10.1186/1471-2458-12-726

**Published:** 2012-08-31

**Authors:** Marie Dauvrin, Ilse Derluyn, Isabelle Coune, Hans Verrept, Vincent Lorant

**Affiliations:** 1 Institute of Health and Society IRSS, Université catholique de Louvain, Clos Chapelle aux Champs 30 boite 1.30.15, 1200, Brussels, Belgium; 2 Fonds de la Recherche Scientifique-FNRS, rue d’Egmont 5, 1000, Brussels, Belgium; 3 Department of Orthopedagogics, Ghent University, Henri Dunantlaan 2, 9000, Gent, Belgium; 4 Intercultural Mediation Cell and Support to the Policy, Ministry of Public Health, Service des soins de santé psychosociaux, SPF Santé publique, Sécurité de la chaîne alimentaire et Environnement, Eurostation bloc II - 1D210, Place Victor Horta 40 boite 10, 1060, Brussels, Belgium

**Keywords:** Ethnic minorities, Health policy, Migrants, Belgium, Quality of care, Access to health care, Health promotion, ETHEALTH

## Abstract

**Background:**

In Europe, progress in the development of health policies that address the needs of migrants and ethnic minorities has been slow. This is partly due to the absence of a strategic commitment by the health authorities. The Ministry of Public Health commissioned the ETHEALTH (**ET**hnicity &**HEALTH**) group to formulate relevant recommendations to the public authorities with a view to reducing health inequalities among ethnic minorities. This paper describes the political process and the outcomes of the ETHEALTH expert group.

**Results:**

After ten meetings, the ETHEALTH group came up with 46 recommendations, which were presented at a national press conference in December 2011. Target groups concerned by these recommendations covered both irregular migrants and migrants entitled to the national insurance coverage. Recommendations were supported by the need of combining universal approaches to health care with more specific approaches. The scope of the recommendations concerned health care as well as prevention, health promotion and access to health care. When analysing the content of the recommendations, some ETHEALTH recommendations were not fully measurable, and time-related; they were, however, quite specific and realistic within the Belgian context. The weak political commitment of an executive agency was identified as a major obstacle to the implementation of the recommendations.

**Conclusions:**

The ETHEALTH group was an example of scientific advice on a global health issue. It also demonstrated the feasibility of coming up with a comprehensive strategy to decrease ethnic health inequalities, even in a political context where migration issues are sensitive. Two final lessons may be highlighted at the end of the first phase of the ETHEALTH project: firstly, the combination of scientific knowledge and practical expertise makes recommendations SMART; and, secondly, the low level of commitment on the part of policymakers might jeopardise the effective implementation of the recommendations.

## Background

Like other European countries, Belgium has to cope with the increasing diversity of its population. Several factors contribute to this diversity, including a long history of labour migration, the country’s colonial past, and, more recently, its strategic position in the European Union [1,2]. Although a certain slowing-down of labour immigration has been observed in recent years, migration flows still persist and asylum applications are still rising.

In Belgium, the health status of migrants and ethnic minority groups (MEM) raises a paradox. On the one hand, some MEM have a lower risk of mortality, lower prevalence of some cancers, and lower alcohol consumption rates compared to Belgians [3]. On the other hand, MEM often experience poorer health status than “native” Belgians: some MEM have more mental health problems, such as psychosis and anxiety, higher rates of chronic diseases, such as type 2 diabetes mellitus, and report poorer subjective health compared to Belgians [4-9]. Moreover, MEM experience linguistic and cultural barriers to access to some health services, such as health promotion facilities, screening services, and specialised care; this can increase their risk of poorer health status because of a lack of (appropriate) care [10,11].

In the last decade, many national and international agencies have placed ethnic health equality higher on the public health agenda. The 2003 US “Unequal Treatment” report and the 2005 Bennett Report in the UK have led to proactive migrant health policies within the health services [12,13]. Since 2000, the European Union has published several directives referring to health care for migrants and required from his members an integration of these directives within the national legislative framework [14-21]. In 2006, the Council of Europe deposed a memorandum on health services in a multicultural society [22]. In 2008 the WHO published Resolution WHA 61.17, highlighting the need to promote migrant-sensitive policies within health systems [23]. This was followed by a policy report of the WHO Regional Bureau for Europe, which advised states on how to address ethnic inequalities [24]. This kind of public advocacy has led to some interesting initiatives, such as the creation of the National Resource Centre for Ethnic Minority Health in Scotland, the development of the Migrant-friendly Hospitals Task Force and the Amsterdam Declaration, and the “Migration and Health” project in Switzerland, led by the Federal Office of Public Health [25-27].

Despite such public advocacy, however, many European countries are still hesitating on how to come up with an explicit commitment in relation to this topic [28]. So far, Belgium has addressed ethnic health inequalities only to a limited extent. Although Belgium had integrated all the European directives aimed at improving the health of MEM into its legislation, its commitment to diversity is rather weak [29-31]. Data on ethnic health inequalities are rather sparse and mostly limited to nationality. Public agencies are prohibited from collecting data on ethnicity [32]. Very few public health policies actually recognise the importance of cultural diversity and the needs of MEM [33]. Most public-health plans (such as the National Plan against Cancer) or blueprints have not paid attention to the particular situation of MEM; this has led to under-provision of health services, prevention, and health promotion [10,11,34]. Health care organisations have no formal obligation to pay attention to diversity, leading to an implicit denial of health care discrimination. Although laws to struggle against racism and discrimination exist, the Centre for Equal Opportunities and Opposition to Racism has an advisory role only and is not considered as an executive agency [35,36]. In addition, within the two most important health executive agencies, there is no strong commitment to improve equity and diversity. Finally, state-funded intercultural mediators are limited to hospitals and there are few interpreting services in outpatient settings.

Recent events have increased the pressure on health policymakers to pay more attention to ethnic health equality in Belgium. Critical incidents – such as the failure to provide asylum applicants with adequate health care or Muslim clients refusing emergency care for gender-based reasons, and epidemiological data on the rising incidence of communicable and non-communicable diseases among MEM – have triggered more interest among public-health stakeholders. Recently, the COST HOME network has put the issue higher on the research agenda in Belgium and in Europe [37]. The recent work of Lorant & Bhopal comparing Belgium and Scotland had highlighted some of the weaknesses of the Belgian health system and prepared the ground to develop a national project [29]. Within this context, the “ETHEALTH” – Ethnicity and Health – expert group was set up to formulate recommendations to the Belgian public health authorities on how to reduce ethnic and migrant health inequalities. This paper describes the process and outcome of elaborating this collaborative strategy to improve equity in health. To our knowledge, this is the first report in continental Europe on how to elaborate a policy blueprint to tackle ethnic inequalities in health. Through this paper, we hope to contribute to the growing debate on changing policies on ethnicity and health as highlighted recently by the COST-ADAPT memorandum [38]. Although there is a growing body of evidence that public-health sciences could contribute to the elaboration of policy, there are few previous experiences of scientific studies producing advice in relation to ethnicity and health in continental Europe [39].

## Methods

### The steering committee

A steering committee was commissioned by one General Director at the Ministry of Public Health. This steering committee was composed of three researchers and two civil servants from the Ministry of Public Health. The steering committee conducted the project, organised the panel meetings, invited the experts, and drafted the report. The steering committee also produced a review of the existing situation in relation to migration and health in Belgium, as well as in other countries, to provide a documentary background for the discussion.

The steering committee invited several experts in the field of health and migration in Belgium to take part in the panel. Experts cited by the five members of the steering committee were contacted and asked to join the core group of ETHEALTH. Four experts refused to participate, either due to lack of time or because they did not consider themselves to be experts. They were replaced by other experts on the list. Twenty-one experts finally constituted the panel. The panel was made up of 8 men and 13 women. Four experts were from migrant backgrounds, 13 experts had French as their preferred national language. Table 1 displays the qualifications and current positions of the ETHEALH experts **[**Table 1**]**.

**Table 1:** Areas of expertise and current positions of the steering committee and the panel group of the ETHEALTH project

	**Area of Expertise**	**Current position**
Expert 1	Health inequalities Medical sociology	Institute of Health and Society, Université catholique de Louvain
Expert 2	Unaccompanied minors Emotional well-being	Department of Orthopedagogics, Ghent University
Expert 3	Health inequalities Cultural Competences	Institute of Health and Society, Université catholique de Louvain
Expert 4	Intercultural mediation Women's health	Intercultural Mediation in Hospitals and Policy Support, Ministry of Public Health
Expert 5	Intercultural mediation Policy support	Intercultural Mediation in Hospitals and Policy Support, Ministry of Public Health
Expert 6	Intercultural mediation Inpatient mental health services	Centre Hospitalier Jean Titeca, Brussels
Expert 7	Undocumented migrants and migrants with a precarious legal status	Steunpunt Gezondheid en Vreemdelingenrecht, Kruispunt Migratie-Integratie
Expert 8	Primary care services Health promotion	Fédération des Maisons Médicales et collectifs de santé francophones
Expert 9	Health promotion Health prevention	Vlaams Instituut voor Gezondheidspromotie en Ziektepreventie (ViGeZ)
Expert 10	Primary care services General practice	University of Antwerp Wijkgezondheidscentra
Expert 11	Equal opportunities in all sectors Legislation and policy	Centre for Equal Opportunities and Opposition to Racism
Expert 12	Women's health and genital mutilation Policy	Ghent University
Expert 13	Social assistance in hospitals Data collection issues	Saint Pierre University Hospital
Expert 14	National Health Interview Survey	Belgian Scientific Institute for Public Health (ISP/WIV)
Expert 15	Intercultural care in primary care services	Foyer asbl/vzw
Expert 16	Access to care for undocumented migrants, social perspective	Doctors of the World
Expert 17	Access to care for undocumented migrants, social and medical perspective	Doctors of the World
Expert 18	Social assistance in hospitals	Centre Hospitalier Universitaire de Charleroi
Expert 19	Privacy regulations	Faculty of Law and Theology, Institut pour la recherche interdisciplinaire en sciences juridiques, Université catholique de Louvain
Expert 20	Social assistance in hospitals Financial issues associated with access to health care	Saint Pierre University Hospital
Expert 21	Transcultural psychiatry Outpatient mental health services	D’Ici et d’Ailleurs asbl/vzw

*Experts with an * are members of the steering committee.

### The panel process and deliverables

Ten panel meetings took place between June 2010 and December 2011. The first meeting agreed on the scope, target group, objectives, and disclosure of the final report. Panel sessions were also devoted to specific topics such as (1) undocumented migrants and asylum seekers; (2) cultural competence, health promotion, and prevention; and (3) monitoring and registration of data on ethnicity in relation to health issues.

At each meeting, participants expressed themselves either in French or Dutch. Simultaneous interpretation was provided by professional interpreters. That service was very important to help avoid misunderstandings, first of all, between French- and Dutch-speaking stakeholders, but also to allow MEM to participate in the process. Based on the transcripts of the meetings, a first version of the report was drafted by the steering committee and sent to the members of the panel group for approval and modification. The ETHEALTH report was structured around the Priority Public Health Conditions Analytical Framework [40]. The final report and recommendations were presented at a national press conference in December 2011. Other dissemination activities were carried out, including communications at national and international conferences. Some dissemination activities were led by the steering group, while others were organised by the members of the ETHEALTH group.

## Results

We report the outcomes concerning the general framework of the debates within the ETHEALTH group, before presenting the recommendations. We then assess how SMART the recommendations are. Finally we analyse the political process.

The target group had to be defined, as the ETHEALTH group acknowledged that “migrants and ethnic minorities” encompass highly heterogeneous groups, with various histories of migration and sometimes very different needs. It was quickly admitted that nationality was no longer an appropriate term, as an important part of the migrant population living in Belgium has Belgian nationality. Accordingly, the ETHEALTH group adopted the wider approach of WHO Europe and consensually decided to use the WHO Europe concept of “Migrants and Ethnic Minorities” (MEM) [24].

Early panel discussions quickly led to a clear distinction between legal MEM entitled to health insurance coverage and irregular MEM, whose health care coverage falls under a different legal regime. The ETHEALTH group felt that these two groups required different policy approaches, although the commissioning agency was initially not that interested in irregular migrants, because that group falls under the responsibility of a different ministry. For public health reasons, however, this group was recognised as highly vulnerable.

A third debate led to an emphasis on the need to combine universal approaches to health care with more specific approaches for MEM. The ETHEALTH group noted that a small number of health care organisations, such as particular public hospitals and primary care surgeries, were increasingly attracting most MEM patients, leading to a *de facto* health care ghetto. In the light of this, the ETHEALTH group urged that all health care systems should be accessible to all population groups, including MEM; this requires that all health care institutions be organised in a “migrant-friendly” way in order to avoid health care ghettos and increase the accessibility and quality of care.

Finally, although its initial commission focused on health care, which is the main competence of the federal Ministry of Public Health, the ETHEALTH group quickly broadened its scope to include access to health care, health promotion, and prevention.

### Recommendations

The ETHEALTH group came up with 46 recommendations [41,42]**[**Table 2]**.** The ETHEALTH report was drafted in line with the Priority Public Health Conditions Analytical Framework [40,43]. This conceptual framework was consensually chosen as an overall model to illustrate the complex interactions between all the recommendations. It includes five interacting levels: context and socio-economic position, differential exposures (risk factors), differential vulnerability (at-risk groups), differential health outcomes, and differential consequences **[**Additional file 1: Figure S1**]**.

**Table 2:** The 46 recommendations of the ETHEALTH group designed to address health inequalities among migrants and ethnic minorities in Belgium

**Topics**	**Recommendations**
**Level 1:Content and socio-economic position**	
(1) Data on MEM	1.1. Identification of migrants and ethnic minorities in systematic health care register
	1.2. Improvement of the statistical power of the National Health Interview Survey for MEM
	1.3. Encouragement of research into MEM health status and health care
2) Coordinating efforts to develop a global and coherent strategy between the different levels of governance	1.4. Improvement of coordination between federal, regional, Community, and municipal levels of governance
	1.5. Encouraging public health authorities to join international networks active in intercultural health care, such as the Migrant- friendly Hospitals network
(3) Training and licensing culturally competent health professionals	1.6. Making cultural competences training a licensing criterion for health professionals
	1.7. Encouragement of the orientation of MEM towards the health professions, to add to the diversity of health care teams
**Level 2: Differential Exposures (risk factors)**	
(1) Reduction of socio-economic inequalities	2.1. Combating labour market discrimination and application of existing legislation in companies
	2.2. Taking into account the specific needs of MEM, especially first-generation, in education, but preventing the creation of educational ghettos and discouraging the systematic orientation of MEM to specialised schools
	2.3. Taking initiatives in several areas to allow the participation of MEM in decisions that concern them
(2) Culturally competent health prevention, health promotion, and health education, including strengthening community health	2.4. Increasing the awareness of health professionals in primary care services of the specific risks experienced by MEM and the higher risk of developing certain diseases, such as tuberculosis, while preventing “ethnification” or “racialisation” of these diseases
	2.5. Structural integration of preventive activities into the existing health care services
	2.6. Adopting proactive initiatives to provide comprehensible and adapted information on the health care system for MEM, with strengthening the role played by the sickness insurance funds in informing clients
	2.7. Considering community health as a main activity of the primary health care services
	2.8. Taking into account, as far as possible, the context of the client in the delivery of health care facilities, especially in chronic treatment and in residential treatment, to avoid dropping out
	2.9. Improvements to the curriculum of community health nurses, in nursing school as well as in the field, and including community health in the agreed standards for primary care services
**Level 3: Differential vulnerability (at-risk groups)**	
(1) Irregular migrants and migrants with a precarious legal status, including asylum-seekers	3.1. Clarifying the application of the legislation on Urgent Medical Aid and ensuring a clear framework of reimbursement for health care for migrants with a precarious legal status
	3.2. Entrusting the Public Centres of Social Action with the social inquiry to decrease the burden on social services in hospitals
	3.3. Delivering to all irregular migrants a voucher entitling them to request assistance from different social and medical institutions
	3.4. Extending the use of the “medical card” to all irregular migrants, entitling them to urgent health care
	3.5. Diversification of the health professionals and health services available to treat MEM with a precarious legal status or in irregular situations, so as to prevent the formation of “health ghettos”
	3.6. Provision of a temporary residence permit for irregular migrants with contagious diseases such as tuberculosis, in order to create confidence and ensure MEM follow a full course of treatment
	3.7. Requiring better support from the Federal Agency for the Reception of Refugees and Asylum Seekers (FEDASIL) for the provision of specific training for these health professionals
	3.8. Ensuring decent reception conditions, that respect human dignity, for all asylum-seekers, to avoid situations where their place of residence may increase mental and physical health problems
	3.9. Ensuring access to all health care services for all asylum-seekers, whatever their conditions of reception/detention
(2) Migrants and ethnic minorities with mental health problems	3.10. Developing and providing culturally competent preventive actions in mental health care, developed in partnership with the target population
	3.11. Developing and providing culturally competent mental health services, especially in urban centres in all the regions of Belgium
	3.12. Developing interpreting facilities and intercultural mediation within mental health care services
	3.13. Improving access to specific training in mental health for health professionals
	3.14. Improving collaborative links and referral between mental health services and associations that assist MEM, whatever their legal status
	3.15. Increasing awareness of mental health problems and adequate referral of clients within the primary care services
(3)Women	3.16. Improving management of MEM women in maternity units, accident and emergency departments, and gynaecological services, with due consideration for cultural, financial, linguistic, or social obstacles
	3.17. Developing diversified responses to the struggle against genital mutilation and sexual violence
	3.18. Improving prevention in relation to sexual and reproductive health for MEM women by improved information on their rights (including abortion, contraception, and protection against conjugal violence)
	3.19. Promoting access to prenatal care and screening services for breast or cervical cancer
**Level 4: Differential health outcomes**	
(1) Reinforcing the accessibility and quality of health services	4.1. Reinforcing the accessibility and organisation of primary care services, especially where needs for intercultural care are more predominant
	4.2. Ensuring free access to health care services and basic drugs for all clients between 0 and 18 years
	4.3. Stimulating a stable relationship with the general practitioner and the creation of a global medical file
	4.4. Stimulating the creation of frontline primary health care centres, working in interdisciplinary teams with integration of social, community, and mental aspects into health care facilities
	4.5. Stimulating the creation of efficient networks involving primary-care services, specialised health care services, support structures in other sectors (labour or housing), and representatives of MEM, in order to ensure the transmission of information and the adequate orientation of clients
	4.6. Providing adequate information to MEM about health and preventive/curative health care services
(2) Promoting culturally competent health care services	4.7. Increasing the accessibility of, and encouraging collaboration with, interpreters and intercultural mediators in all health services
	4.8. Increasing awareness of culturally specific components in health care delivery, with a view to improving the accessibility and quality of health care for MEM (e.g. adaptation of meals to religious precepts or religious facilities inside the institution)
	4.9. Stimulating the openness and awareness of health professionals to diagnosis and management of MEM
	4.10. Promoting collaborative work with diversified health care teams (gender, age, culture, or educational level)
	4.11. Encouraging each health professional and each health service to develop action plans and to mobilise resources to meet the needs of MEM

At the contextual and socio-economic levels, the ETHEALTH group made the case for better data on MEM, improved coordination across the different levels of governance, and making cultural competences a licensing criterion for all health care professionals. For example, Belgium already has a National Health Survey by interview, but there is no recording of the ethnic background of the participants. An additional sample of MEM might provide more reliable estimates of the health of MEM living in Belgium (recommendation 1.2).

In order to decrease differential exposure, the ETHEALTH group recommended improving equal socio-economic opportunities for MEM, fighting against discrimination, and improving preventive health care among MEM. For example, the ETHEALTH group recommended the integration of preventive activities into the primary health care services: these activities must be relevant to the neighbourhood where the health service is located (recommendation 2.5).

In relation to differential vulnerability, the ETHEALTH group identified three more at-risk groups among MEM: irregular migrants and asylum seekers, MEM with mental health problems, and women. These three groups have several risk factors for having a poorer health status than the “native” population and experiencing discrimination due to the multiplication of risks. For example, in order to reduce administrative procedures and facilitate access to health services, the ETHEALTH group supported the generalisation of the “medical card” for irregular migrants. In areas where it already exists, the medical card for irregular migrants gives access to health care services for a particular period, without additional administrative procedures. For MEM with mental health problems, the ETHEALTH group advised an improvement in collaboration, including referrals of patients, between mental health services and supportive associations for MEM, such as community associations (recommendation 3.14). For women, the ETHEALTH group recommended an improvement of the management of MEM women in maternity wards, accident and emergency departments, and gynaecological services. Using interpreters, in order to avoid relying on interpretation by husbands, or cultural mediators, in order to make less likely the refusal of care by women (or their families) because of gender issues, are two concrete initiatives that could reduce the isolation of some MEM women (recommendation 3.16).

In relation to health outcomes, the ETHEALTH group suggested improving the quality and accessibility of health care. Several existing initiatives were described in the report [41,42]. One interesting recommendation is to improve the development of intercultural mediation, which already exists in hospitals but is lacking in GP surgeries (recommendation 4.7).

#### Are the ETHEALTH recommendations S.M.A.R.T?

We also assess how SMART these recommendations are, with regard to the following criteria: Specificity, Measurability, Assignability, degree of Realism, and Time-relatedness [44].

### Specificity: universal versus specific approaches

Fourteen recommendations could be considered as universal, while 32 are specific recommendations for MEM. Among the specific recommendations, 18 concern three more vulnerable groups: irregular migrants (9 recommendations), women (3 recommendations), and MEM with mental health problems (6 recommendations). Because of specific risk factors, these three groups are considered to be at greater risk of having severe health problems or experiencing exclusion and discrimination. Universal recommendations mainly concern improvements in the accessibility and quality of care for the entire population, and not specifically for MEM. For example, recommendation 4.2 argues for free access to primary health care for all children and young people below the age of 18.

### Measurability

A recommendation is measurable when it is possible to quantify it or at least when an indicator of progress is suggested. Actually, none of the recommendations has an indicator of progress or a measurable target. In some cases, however, the recommendation suggests a concrete action, whose measurement does not imply a quantitative measurement: this is the case with recommendation 1.2, which argues for a better sampling of MEM in the National Health Interview Survey. Moreover, recommendations 3.1 to 3.6 are extremely concrete and their adoption is likely to lead to explicit outcomes. For example, recommendation 3.6 suggests granting a temporary residence permit to irregular migrants with a contagious disease such as tuberculosis, in order to create confidence and ensure MEM follow a full course of treatment.

### Assignability: who are the recipients?

The ETHEALTH recommendations target several stakeholders. Twenty-three recommendations target political actors (e.g. recommendations 1.4, 3.1, 3.6, and 4.1): these may concern federal public authorities or regional public authorities. A total of 36 recommendations directly concern the health care sector, while 10 are addressed to non-health sectors such as the labour market (see, e.g., recommendations 2.1, 2.2, and 2.3). Finally, 23 recommendations require adaptations by health professionals and the health services (see, e.g., recommendations 3.14, 3.17, 4.3, and 4.5) and no recommendation targeted the clients. Recommendations should also assign recommendation implementation to one or several agencies. However, we notice that none of the recommendations identifies the agencies responsible for implementation.

### Degree of realism: what are the changes required?

Broadly, the recommendations require marginal changes. We identify three levels of change in the ETHEALTH recommendations: marginal change, enforcement of existing regulations, and radical change. Firstly, 23 recommendations require only marginal changes; these recommendations do not require legal changes or a new distribution of power. Moreover, the climate is already favourable for the implementation of some of these recommendations. Recommendation 1.6, for example, argues for the inclusion of cultural competency training in the licensing requirements for health professionals. There is currently a reform of the medical curriculum under way in Belgium that creates some momentum for the introduction of new skills into the education of medical doctors. Secondly, 14 recommendations involve the implementation of existing regulations and go on to argue for improvements in the application of these rules, for example, those that are intended to combat discrimination on the labour market (recommendation 2.1). Thirdly, 9 recommendations require more radical changes. For example, recommendation 4.2 argues for free health care for children between 0 and 18 years of age; this would require a modification of health insurance coverage, funding rules, and legislation.

### Time-relatedness

None of the recommendations has a deadline for implementation. Uncertainty about when the recommendations would be published may have played a role here.

#### Assessment of the political process

We now describe the role of the policy process in adopting and – possibly – implementing these recommendations within the “Shiffman and Smith” framework [45]. This framework is helpful when it comes to anticipating difficulties in implementing the recommendations and the resources needed to overcome those difficulties: the cohesion of the political community, the ideas portraying the problem, the political context, and the issue importance.

In relation to the cohesion of the political community, ETHEALTH group succeeded in drawing up inter-sectorial recommendations in a context of considerable political and administrative fragmentation within Belgium, where ethnicity remains a relatively neglected topic. The heterogeneity of the experts contributed to the development of a wide range of recommendations. These recommendations concerned the individual level as well as the institutional and political levels. However, implementation may still be endangered because of the high level of political, legal, and administrative fragmentation applying to health and ethnicity in Belgium. The most obvious fragmentation has to do with devolution and the North–south divide, which was particularly burning at the time. We noticed, for example, that French-speaking representatives were less keen to attend the meetings than Dutch-speaking representatives. Press coverage was also somewhat less favourable in the French-speaking media. Moreover, although the Ministry of Public Health commissioned ETHEALTH, the political context was not favourable to strong commitment: no key figure emerged to make a public commitment to the implementation of the recommendations. The “Centre for Equal Chances and Opposition to Racism” has a mandate that is somewhat closer to the objectives of ETHEALTH, but it has only an advisory role in the Belgian statutory set-up. In fact no guiding institution with a definite executive mandate currently exists in Belgium to cope with these issues. This may also jeopardise the implementation of the recommendations, as issues of ethnicity and health cut across many institutions. The absence of policymakers did, however encourage open and transparent discussion and no partisan affiliation was necessary to participate. Finally, the publication of the ETHEALTH group report led the Ministry of Public Health to call for an inter-ministerial conference, a code name in Belgium for involving federal and regional policymakers. Such conferences lay the foundations for cooperation between federal and regional authorities in order to promote more coherent planning. The ETHEALTH group also acknowledged the need to adapt the recommendations according to institutional responsibilities.

Given the complexity and variety of the topics involved, the ETHEALTH group had to structure the recommendations using a wide spectrum of ideas. The steering group suggested the Priority Public Health Conditions Analytical Framework as a framework for organising the diverse recommendations and preserving the multiple perspectives highlighted by the experts [43]. In that sense, ETHEALTH represents a valuable application of this framework to specific vulnerable groups. The Priority Public Health Conditions Analytical Framework was seen as a relevant model for a structured approach to broad issues such as migration and health.

The political context was not favourable: the low level of political commitment beforehand and the absence of a federal government at the time made the whole project risky. ETHEALTH group could not assume that its recommendations would have an immediate impact and was aware that its recommendations might not be implemented. This particular situation influenced the debates and the outcomes. It was clear at the time that health care would be part of the institutional reshuffle that was expected to lead to a new devolution of responsibilities to regional authorities, which were not sitting around the table. A change in the political orientation of the Ministry of Public Health may also have impeded the diffusion of the results. Finally, the new federal government was formed just two weeks before the press conference. The day the results were presented, the federal government announced the reform of the pension system: as a consequence, the ETHEALTH recommendations were downgraded to a lower priority level both inside the government and in the media.

Finally, the importance attributed to these issues was difficult to estimate, mainly because of data scarcity. Although the preliminary review of the existing data highlighted the importance of these issues, additional data are needed on migrant health in Belgium. Consequently, the first ETHEALTH recommendation concerns the need to record data on ethnic inequalities (see recommendation 1.1).

## Discussion

The ETHEALTH recommendations were specific and, broadly, realistic within the Belgian context: they were mostly based on existing Belgian experience. Most of the recommendations require marginal changes, meaning that the potential to develop fair policies for MEM already exists in Belgium. Some recommendations were clearly defined and had a clear audience. This was particularly the case for recommendations 3.1 to 3.6, which had been discussed for a long time before ETHEALTH group was set up by a forceful advocacy group, “Kruispunt Migratie Integratie”, on behalf of irregular migrants. This example confirms the importance of having a lead agency in the field of migration and health in order to sustain the development of migrant-friendly policies.

Uncertainty, however, remains when it comes to translating these recommendations into practice. The ETHEALTH recommendations were not all fully SMART (Specific, Measurable, Assignable, Realistic, and Time-related). They were not formally assigned to specific stakeholders, although they suggested implicit assignment. Lack of assignation of the recommendations may lead to a dilution of responsibilities among stakeholders, with minimal commitment to implementation. Indeed, at the time, the political instability did not favour such assignability: the government had no mandate to make strong commitments and the devolution of some responsibilities to regional authorities was under discussion. Moreover, ETHEALTH group had no explicit mandate to assign the recommendations to public authorities. Similarly, the recommendations did not suggest a time schedule for implementation or indicators to assess the implementation of the recommendations. Developing an implementation plan requires a lead agency and resources to achieve it; in the field of migrant health, it is currently hard to see which agency this might be.

Are the ETHEALTH recommendations consistent with similar achievements in the same field? Recently, the Council of Europe published recommendations on mobility, migration, and access to health care [46]. It turns out that the content and structure of the ETHEALTH recommendations were similar to the recommendations of the Committee of Ministers of the Council of Europe. The recommendations of the Council of Europe did not establish a deadline for achievement, nor were there suggested indicators of progress, but the recommendations were specific and realistic at the European level. In the Scottish “Fair for All” policy and in the Amsterdam Declaration, recommendations are quite specific but do not address inequalities within the general population. The reduction of health inequalities appears to be more efficient when generic and specific interventions are combined in order to improve the health status of the whole population. Therefore, these interventions require an evaluation of their impact that takes into account the gender, ethnicity, and socio-economic status of different population groups [47]. On the other hand, the Scottish “Fair for All” recommendations were highly measurable, with clear indicators of achievement; they were realistic and had a time schedule for implementation according to the efforts the services had to make. We also noticed that these Scottish recommendations were assigned only to the managers of the health services and health professionals [26]. Similarly, in Switzerland, the federal strategy for 2008–2013 suggested specific, time-related recommendations to improve health equity [48]. The Swiss recommendations were assignable and spread across several levels, like the ETHEALTH recommendations. They were highly realistic and a specific budget was set out. But one of the main differences between the ETHEALTH recommendations and the Swiss and Scottish programmes is the initiator of the policy review in question. In both Scotland and Switzerland the public authorities were the source of the project. Specific legislation against discrimination, such as the UK Race Relations (Amended) Act of 2000, already existed, putting pressure on the authorities to develop diversity plans, including in the health services. The commissioning of ETHEALTH by the Ministry of Public Health was in part triggered by the scientific community.

ETHEALTH looked at a very broad spectrum, including first-generation migrants and ethnic groups, insured and non-insured migrants, and health problems, as well as upstream factors such as socio-economic inequalities or health care coverage. This differs, however, from previous European policies on migrant health. Indeed, as previously shown, most European policies tend to focus on first-generation migrants or on ethnic minorities, whereas ETHEALTH looks at all migrant groups [49,50].

The study had some limitations. Members of the ETHEALTH group were selected on a voluntary basis. Only 4 experts belong to an ethnic minority group and no representative of users was invited. As the MEM represent a highly heterogeneous population in Belgium, the ETHEALTH group decided against inviting users' representatives, in order to avoid an over- or underestimation of some groups in the discussion. However, the preparatory review of the existing data also looked at studies of the needs and expectations of MEM, with a view to including these in the recommendations. In future phases, ETHEALTH might consider organising discussions with representatives of users and representatives of the MEM communities, such as the Muslim Executive, in order to validate the recommendations and tailor them to the needs of clients.

We also noticed that most of the experts on the panel had a patient-centred perspective on health care. None of the health experts was believed to be active in a “biomedicine” service. Experts with a patient-centred perspective may be more favourably disposed to the adaptation of health care to cultural diversity than experts without a patient-centred perspective [50]. Some skills and expertise were lacking: further projects could also involve discussion and validation of the recommendations with representatives of medical and paramedical disciplines.

## Conclusions

The ETEHALTH group was an example of “science-advising” on a global health issue. It also demonstrated the feasibility of coming up with a comprehensive strategy to decrease ethnic health inequalities, even in a political context where migration issues are sensitive. The second positive outcome of ETHEALTH has been to create a stronger community of both researchers and field workers; this may help to bring together practical experience and scientific expertise and improve the cohesion of the community.

Two final lessons may be highlighted at the end of the first phase of the ETHEALTH project. Firstly, the topic for which the recommendations were the most SMART was the one for which both scientific knowledge and practical expertise were available. Secondly, a lack of commitment of policymakers might jeopardize effective implementation.

## Endnotes

^a^Levels displayed within the Table 2 are based on the Priority Public Health Conditions Analytical Framework [43] (Additional file 1: Figure S1).

^b^In the recommendation 3.4 (Table 2), the « medical card » is not the national health insurance card. It is a special card entitling the bearer to urgent health care.

## Abbreviations

ETHEALTH (acronym): Ethnicity and Health; SMART (acronym): Specific, Measurable, Assignable, Realist and Tim-related; MEM: Migrants and Ethnic Minorities; US: United States of America; UK: United Kingdom; WHO: World Health Organisation; CSDH: Commission on Social Determinants of Health; COST HOME (acronym): European Cooperation in Sciences and Technologies–Health and Social Care for Migrants and Ethnic minorities in Europe network.

## Competing interests

The authors declare that they have no competing interests.

## Authors’ contributions

MD carried out the analysis of the findings of the ETHEALTH group, contributed to the background review of ETHEALTH project and drafted the manuscript. VL helped to draft the manuscript and contributed to the analysis of the findings. ID and VL chaired the debates and revised the manuscript. IC and HV revised the manuscript. All authors contributed to the redaction of the ETHEALTH report and gave their approval to the final version of the manuscript.

## Pre-publication history

The pre-publication history for this paper can be accessed here:

http://www.biomedcentral.com/1471-2458/12/726/prepub

## Supplementary Material

Additional file 1 **Figure S1.** Priority Public Health Conditions Analytical Framework retrieved from Blas & Sivasankara Kurup (eds) 2010, page 7 [43] (figure reproduced with the amiable autorisation of the World Health Organisation) © World Health Organisation 2010. Click here for file
