# Supplementary material for: Towards fair health policies for migrants and ethnic minorities: the case-study of ETHEALTH in Belgium
Source: BMC Public Health. 2012 Aug 31;12:726. doi: 10.1186/1471-2458-12-726 (PMC3520724; doi:10.1186/1471-2458-12-726)
Supplement: Additional file 1 — Figure S1. Priority Public Health Conditions Analytical Framework retrieved from Blas & Sivasankara Kurup (eds) 2010, page 7 [43] (figure reproduced with the amiable autorisation of the World Health Organisation) © World Health Organisation 2010. [file 1471-2458-12-726-S1.pdf]

Table 2 Areas of expertise and current positions of the steering committee and the panel group of the ETHEALTH project

Table 2.a. Areas of expertise and current positions of the members of the ETHEALTH steering committee

	**Area of Expertise**	**Current position**
Expert 1	Health inequalities<br>Medical sociology	Institute of Health and Society, Université catholique de Louvain
Expert 2	Unaccompanied minors<br>Emotional well-being	Department of Orthopedagogics, Ghent University
Expert 3	Health inequalities<br>Cultural Competences	Institute of Health and Society, Université catholique de Louvain
Expert 4	Intercultural mediation<br>Women's health	Intercultural Mediation in Hospitals and Policy Support, Ministry of Public Health
Expert 5	Intercultural mediation<br>Policy support	Intercultural Mediation in Hospitals and Policy Support, Ministry of Public Health

Table 2.b. Areas of expertise and current positions of the experts on the ETHEALTH panel group

	**Area of Expertise**	**Current position**
Expert 6	Intercultural mediation<br>Inpatient mental health services	Centre Hospitalier Jean Titeca, Brussels

Expert 7	Undocumented migrants and migrants with a precarious legal status	Steunpunt Gezondheid en Vreemdelingenrecht, Kruispunt Migratie-Integratie
Expert 8	Primary care services<br>Health promotion	Fédération des Maisons Médicales et collectifs de santé francophones
Expert 9	Health promotion<br>Health prevention	Vlaams Instituut voor Gezondheidspromotie en Ziektepreventie (ViGeZ)
Expert 10	Primary care services<br>General practice	University of Antwerp<br>Wijkgezondheidscentra
Expert 11	Equal opportunities in all sectors<br>Legislation and policy	Centre for Equal Opportunities and Opposition to Racism
Expert 12	Women's health and genital mutilation<br>Policy	Ghent University
Expert 13	Social assistance in hospitals	Saint Pierre University Hospital
Expert 14	National Health Interview Survey<br>Data collection issues	Belgian Scientific Institute for Public Health (ISP/WIV)
Expert 15	Intercultural care in primary care services	Foyer asbl/vzw
Expert 16	Access to care for undocumented migrants, social perspective	Doctors of the World
Expert 17	Access to care for undocumented migrants, social and medical perspective	Doctors of the World

Expert 18	Social assistance in hospitals	Centre Hospitalier Universitaire de Charleroi
Expert 19	Privacy regulations	Faculty of Law and Theology, Institut pour la recherche interdisciplinaire en sciences juridiques, Université catholique de Louvain
Expert 20	Social assistance in hospitals<br>Financial issues associated with access to health care	Saint Pierre University Hospital
Expert 21	Transcultural psychiatry<br>Outpatient mental health services	D’Ici et d’Ailleurs asbl/vzw
